# The correlation between *CRB1* variants and the clinical severity of Brazilian patients with different inherited retinal dystrophy phenotypes

**DOI:** 10.1038/s41598-017-09035-1

**Published:** 2017-08-17

**Authors:** Fabiana Louise Motta, Mariana Vallim Salles, Karita Antunes Costa, Rafael Filippelli-Silva, Renan Paulo Martin, Juliana Maria Ferraz Sallum

**Affiliations:** 1 Department of Ophthalmology, Federal University of Sao Paulo, Sao Paulo, Brazil; 2 Department of Biophysics, Federal University of Sao Paulo, Sao Paulo, Brazil

## Abstract

Inherited retinal dystrophies are characterized by progressive retina degeneration and mutations in at least 250 genes have been associated as disease-causing. *CRB1* is one of many genes analyzed in molecular diagnosis for inherited retinal dystrophy. Crumbs homolog-1 protein encoded by *CRB1* is important for cell-to-cell contact, polarization of epithelial cells and the morphogenesis of photoreceptors. Pathogenic variants in *CRB1* lead to a huge variety of phenotypes ranging from milder forms of inherited retinal dystrophy, such as retinitis pigmentosa to more severe phenotypes such as Leber congenital amaurosis. In this study, seven novel likely-pathogenic variants were identified: four missense variants (p.Leu479Pro, p.Ala921Pro, p.Cys948Arg and p.Asp1031Asn), two frameshift deletions (c.2536_2542del7 and c.3460_3461delTG) and one frameshift indel variant (c.276_294delinsTGAACACTGTAC). Furthermore, two patients with cone-rod dystrophy due to mutations in *CRB1* were reported, supporting previous data, in which mutations in *CRB1* can also cause cone-rod dystrophy. Finally, our data suggested there was a direct relation between phenotype severity and the mutation effect on protein functionality in 15 Brazilian *CRB1* patients.

## Introduction

The *CRB1* gene is associated with some inherited retinal dystrophies (IRD). In humans, it is located on chromosome 1q31.3, composed of 12 exons and encodes a protein with 1406 amino acids, called Crumbs homolog-1. This protein participates in a conserved protein network involved in the morphogenesis of photoreceptors and the establishment and maintenance of apico-basal polarization and adherent junctions of epithelial cells^1–3^.

Crumbs homolog-1 is in a subapical region of photoreceptors, it has a large extracellular part composed of 19 epidermal growth factor (EGF)-like domains and 3 laminin A globular (AG)-like domains, one transmembrane segment and a small cytoplasmic domain. The intracellular domain has a juxtamembrane FERM-binding motif and a carboxy-terminal PDZ-binding motif, by means of which CRB1 interacts with other proteins forming a complex that participates in adherent junction formation and links to cytoskeletons^3, 4^.

Mutations in *CRB1* lead to retinal abnormalities such as thickening, coarse lamination patterns and loss of photoreceptor signalling^1^. Currently, more than 200 mutations in *CRB1* have been cited in the Human Gene Mutation Database - HGMD^5^. The main diseases caused by mutations in *CRB1* are: retinitis pigmentosa (RP) either with or without paraarteriolar preservation of retinal pigment epithelium (PPRPE), Leber congenital amaurosis (LCA) and pigmented paravenous chorioretinal atrophy^6, 7^.

In this study, a large number of medical records of IRD Brazilian patients were reviewed, where 15 patients with CRB1 mutations were selected, and two of them presented cone-rod dystrophy (CRD). Seven new disease-causing variants were reported and a direct relation between phenotype severity and the impact on protein functionality caused by mutation was observed.

## Results

Among the 230 medical records of IRD patients analyzed, 15 cases of unrelated patients with *CRB1* variants were selected, where 13 of them had conclusive molecular diagnosis, whereas in the other two, only one variant was found, presenting a non-conclusive molecular diagnosis. All 15 patients received clinical diagnoses, wherein eight of them were diagnosed as LCA, three as RP, two as CRD and two as early-onset retinal dystrophy (EORD).

### Clinical findings

**Figure 1:**
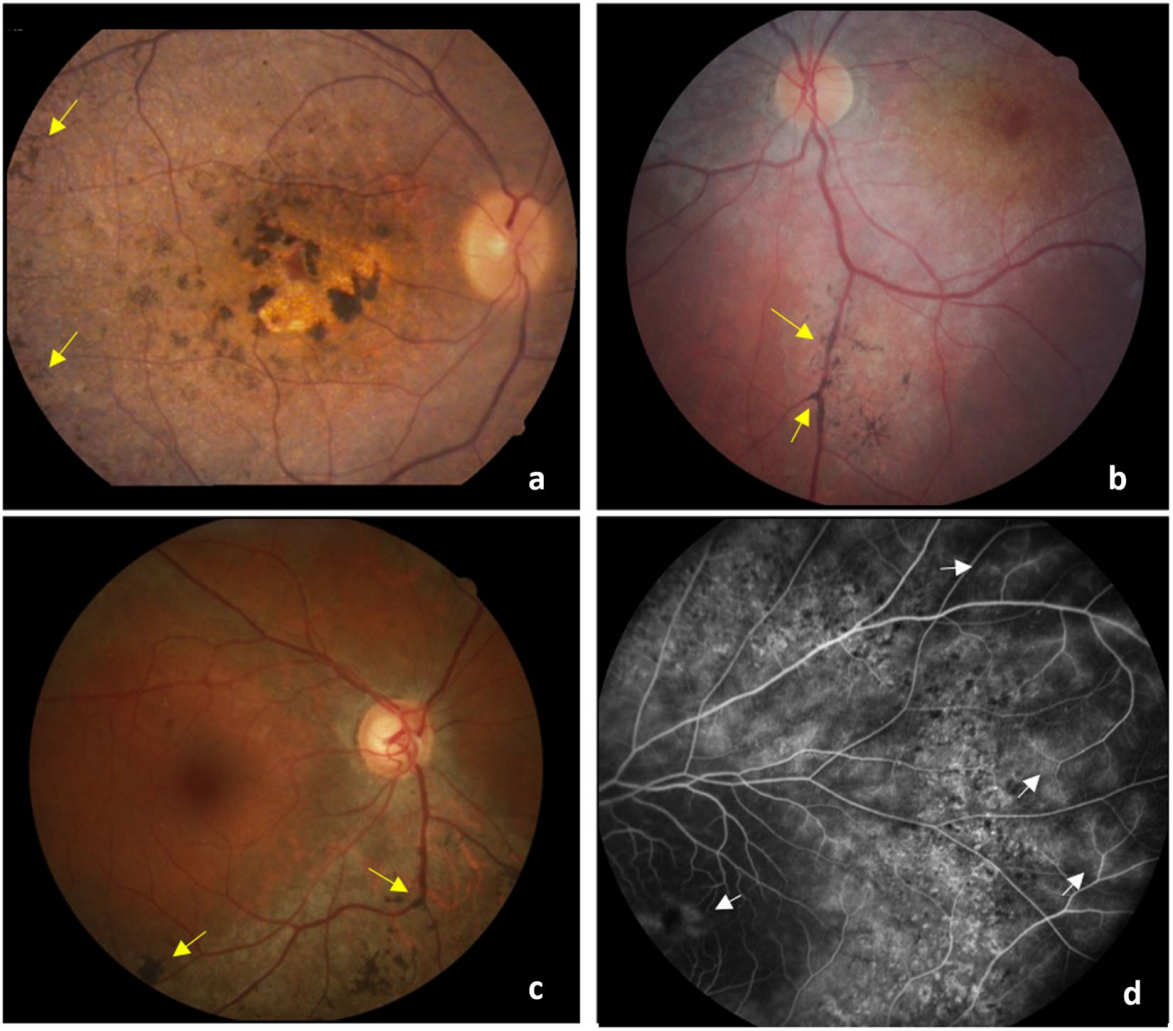
Fundus appearance from *CRB1* patients. (**a**) Color fundus photograph of LCA patient showing the nummular pigmentation and macular atrophy. (**b**) Color fundus photograph of CRD patient showing macular atrophy. (**c**) Color fundus photograph of RP patient showing RPE atrophy and macular area perverted. (**d**) Fluorescein Angiography with fluorescein leakage in peripheral vessels and at the macula. Yellow arrows indicate bone spicules and white arrows indicate leakage of fluorescein.

All eight patients with LCA exhibited the initial symptoms before the first year after birth. Low vision and nystagmus were the most striking features of this group. Only patient 5 did not present nystagmus. Visual acuity of LCA patients ranged from: reduced vision (patient 1) to severe visual loss (patient 4) (Table 1). The typical nummular pigmentation and macular atrophy could be observed from the fundus photographs. In some of them, there was a yellow deposit present in the macular area and widespread white dots in the retinal pigment epithelium (RPE) (Fig. 1a and Supplementary Figure S1).

**Table 1:** Clinical Data of *CRB1* patients.

Patient	Signs and Symptoms	Onset of First Symptoms	Age at time of Diagnosis	Visual Acuities (OD; OS)	Clinical Diagnosis
1	Nystagmus; Reduced visual acuity improved with the development of patient.	first year of life	27	20/60; 20/100	LCA
2	Nystagmus	since birth	6 months	good fix and follow behavior	LCA
3	Nystagmus; Deep reduced visual acuity; mild enophthalmos.	since birth	27	20/1600; 20/1600	LCA
4	Nystagmus; Severe visual loss; Minimum residual temporal visual field in the right eye; Divergent strabismus in the left eye.	first year of life	20	Counting fingers	LCA
5	Non-Nystagmus; Reduced visual acuity; Intermittent exotropia	since birth	7	20/200; 20/200	LCA
6	Nystagmus	3 months of life	3	hand movements perception	LCA
7	Nystagmus; Sub-normal vision	2 months of life	2	20/200; 20/200	LCA
8	Nystagmus; Progressive reduced visual acuity	first year of life	16	20/80; 20/50	LCA
9	Non-Nystagmus; Tubular visual field; Strabismus	5 years old	10	20/60; 20/60	EORD
10	Non-Nystagmus; Reduced visual acuity; Nyctalopia	6 years old	12	20/400; 20/400	EORD
11	Non-Nystagmus in the beginning; Nyctalopia	9 years old	9	temporal perception of light and light movement	CRD
12	Non-Nystagmus; Reduced central visual acuity.	7 years old	24	20/200; 20/400	CRD
13	Non-Nystagmus; Reduced visual acuity even with glasses; Tubular visual field; Nyctalopia	adolescence	18	20/80; 20/80	RP
14	Non-Nystagmus; Convergent strabismus; Hearing loss; Myopia; Glaucoma; Tubular visual field; Nyctalopia	adolescence	47	20/40; 20/25	RP
15	Non-Nystagmus; Tubular visual field; Nyctalopia	adulthood	59	20/20; 20/30	RP

Four patients had showed the first signs and symptoms since their childhood. No nystagmus was present in any of them. Patient 9 with EORD had peripheral vision impairment (tunnel vision) with nummular pigmentation in the RPE and, patient 10 had a central vision impairment, midperiphery with bone spicules and granular pigmentation in the RPE (Supplementary Figure S2). On the other hand, patients diagnosed with CRD (11 and 12) had a more severe impairment of central vision (Table 1) and the fundus examination showed bone spicules with perivascular pattern, as well as macular atrophy characteristics of CRD (Fig. 1b and Supplementary Figure S2). In addition, patient 12 presented an atypical fundus pattern for cone-rod dystrophies, with a well-delimited hyperfluorescent area (Supplementary Figure S3).

As expected for the RP group, the first signs and symptoms appeared either during adolescence or later, and the absence of nystagmus was common in all, with visual acuity 20/80 or less (Table 1). Fundus analysis showed macular preservation compatible with their visual acuity, granular pigments in the RPE and the peripheral presence of bone spicules (Fig. 1c and Supplementary Figure S2). Only patient 13 had RP with PPRPE.

In relation to vascular aspects, patients 2, 4, 6 and 9 showed increased vascular tortuosity. The increased vascular permeability compatible with Coats-like disease onset was noted in two patients with LCA (patient 1 and 8), two with RP (patients 13 and 15), one with EORD (patient 10) and another with CRD (patient 11). Leakage of fluid and blood in Coats-like diseases usually occurs in peripheral vessels, but it may also occur in the macula, causing cystoid macular edema, as observed in patient 15 (Fig. 1d).

### Genetic findings

Table 2 shows the genotypes of patients in this study. All presented variants are classified as pathogenic according to HGMD^5^, except the new variants, highlighted in bold. Patients 14 and 15 did not have a conclusive molecular result because the second pathogenic *CRB1* variant was not found. Ten patients of the 13 genetically concluded cases are compound heterozygotes, whereas the remaining three are homozygotes (patients 3, 8 and 11). In addition, patient 11 is descended from a consanguineous marriage.

**Table 2:** Genotypes of patients with *CRB1* variants.

Patient	Allele 1		Allele 2		Clinical Diagnosis
	Nucleotide Change	Protein Change	Nucleotide Change	Protein Change	
1	c.2843 G > A	p.Cys948Tyr	c.3676 G > T	p.Gly1226*	LCA
2	**c.2536_2542del7**	p.Gly846Serfs*8	c.2843 G > A	p.Cys948Tyr	LCA
3	c.984 G > A	p.Trp328*	c.984 G > A	p.Trp328*	LCA
4	**c.2536_2542del7**	p.Gly846Serfs*8	c.2843 G > A	p.Cys948Tyr	LCA
5	c.984 G > A	p.Trp328*	c.2843 G > A	p.Cys948Tyr	LCA
6	**c.2842** **T** **>** **C**	**p.Cys948Arg**	c.2843 G > A	p.Cys948Tyr	LCA
7	**c.2842** **T** **>** **C**	**p.Cys948Arg**	**c.3460_3461delTG**	p.Cys1154*	LCA
8	c.2843 G > A	p.Cys948Tyr	c.2843 G > A	p.Cys948Tyr	LCA
9	c.2291 G > A	p.Arg764His	c.4168 C > T	p.Arg1390*	EORD
10	**c.276_294delinsTGAACACTGTAC**	p.Arg92Serfs*54	c.2506 C > A	p.Pro836Thr	EORD
11	**c.1436** **T** **>** **C**	**p.Leu479Pro**	**c.1436** **T** **>** **C**	**p.Leu479Pro**	CRD
12	**c.2761** **G** **>** **C**	**p.Ala921Pro**	**c.3091** **G** **>** **A**	**p.Asp1031Asn**	CRD
13	c.2506 C > A	p.Pro836Thr	c.3320 T > G	p.Leu1107Arg	RP
14	c.498_506del9	p.Ile167_Gly169del	not found	not found	RP
15	c.614 T > C	p. Ile205Thr	not found	not found	RP

The novel variants are indicated in bold.

Interestingly, all LCA subjects have more severe pathogenic variants in both alleles. These variants cause premature termination or structural change of the protein due to loss of cysteines involved in disulfide bond formation. In our study, two cysteines forming disulfide bonds are affected: Cys948 and Cys1154. On the other hand, RP and CRD patients have mostly missense variants that do not affect cysteines involved in disulfide bonds. The only exception was patient 14, who has one in-frame deletion with loss of three amino acids (Ile-Asp-Gly), which does not induce formation of a premature stop codon.

**Figure 2:**
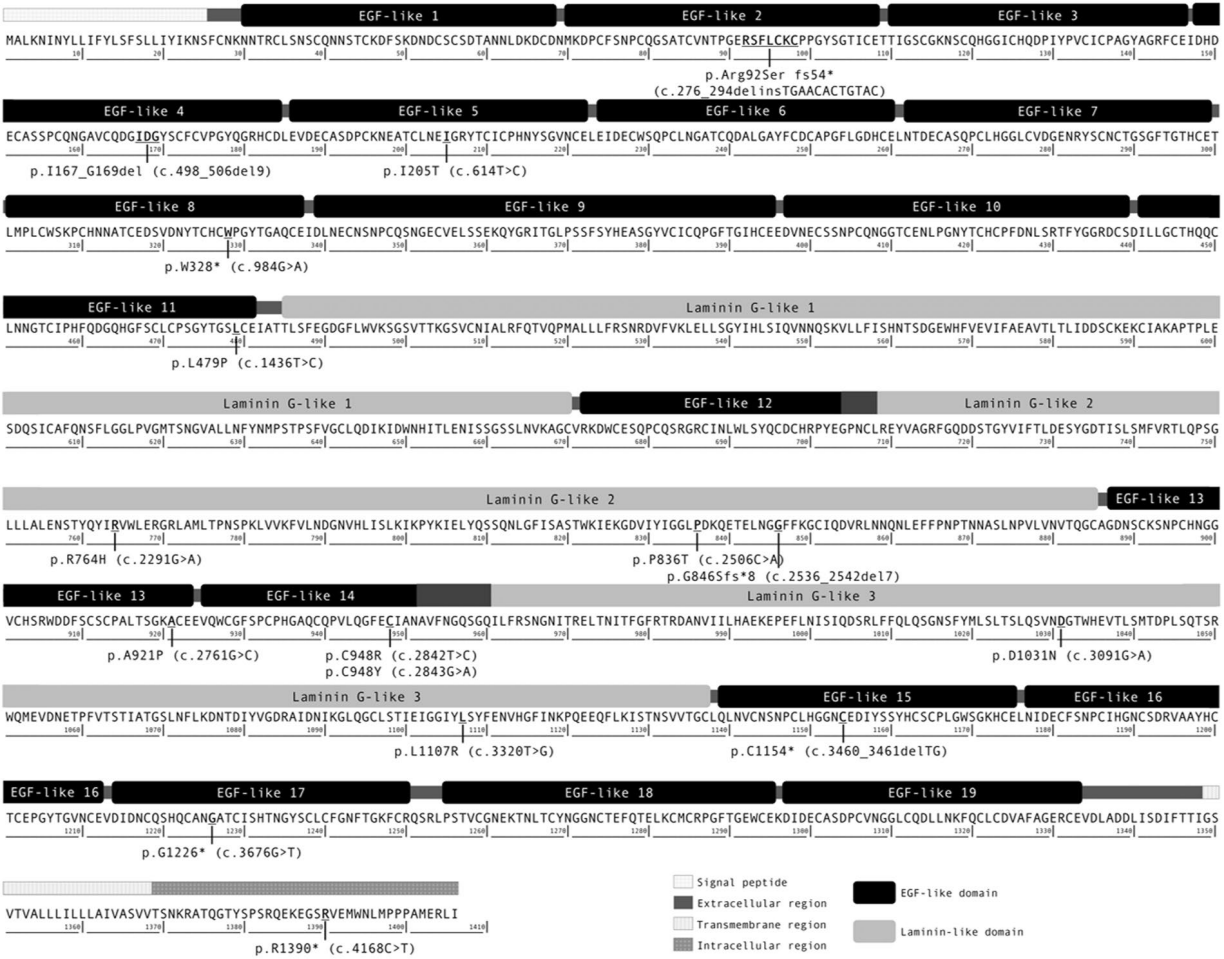
Distribution of *CRB1* variants in the protein.

Sixteen different variants in *CRB1* were found in these subjects. All of them are located in protein domains (Laminin(AG)-like or EGF-like domains) in crumbs homolog-1 extracellular segment, the only exception was p.Arg1390* variant that is in the cytoplasmic domain (Fig. 2). The variants found in this study are preferably located in exons 2, 7 and 9 (three variants at exon 2 and 7 and five variants at exon 9). p.Cys948Tyr was the most frequent in this study (7 alleles in 30 analyzed) (Table 3).

**Table 3:** Variants Data.

*CRB1* Variant	Exon	Protein Region	Protein Domain	Reported phenotype in HGMD (accession)	Allele Frequency^†^
c.276_294delinsTGAACACTGTAC (p.Arg92Serfs*54)	2	Extracellular	EGF 2	not reported	1/30
c.498_506del9 (p.Ile167_ Gly169del)	2	Extracellular	EGF 4	LCA, RP, Stargardt (CD061397)	1/30
c.614 T > C (p. Ile205Thr)	2	Extracellular	EGF 5	RP, LCA (CM033359)	1/30
c.984 G > A (p.Trp328*)	4	Extracellular	EGF 8	LCA (CM1310165)	3/30
c.1436 T > C (p.Leu479Pro)	6	Extracellular	EGF 11	not reported	2/30
c.2291 G > A (p.Arg764His)	7	Extracellular	Laminin AG 2	RP (CM130791)	1/30
c.2506 C > A (p.Pro836Thr)	7	Extracellular	Laminin AG 2	RP (CM043271)	2/30
c.2536_2542del7 (p.Gly846Serfs*8)	7	Extracellular	Laminin AG 2	not reported	2/30
c.2761 G > C (p.Ala921Pro)	8	Extracellular	EGF 13	not reported	1/30
c.2842 T > C (p.Cys948Arg)	8	Extracellular	EGF 14	not reported	2/30
c.2843 G > A (p.Cys948Tyr)	9	Extracellular	EGF 14	RP, LCA, EORD (CM992152)	7/30
c.3091 G > A (p.Asp1031Asn)	9	Extracellular	Laminin AG 3	not reported	1/30
c.3320 T > G (p.Leu1107Arg)	9	Extracellular	Laminin AG 3	LCA (CM057656)	1/30
c.3460_3461delTG (p.Cys1154*)	9	Extracellular	EGF 15	not reported	1/30
c.3676 G > T (p.Gly1226*)	9	Extracellular	EGF 17	LCA (CM113150)	1/30
c.4168 C > T (p.Arg1390*)	12	Cytoplasmic	none	RP (CM130803)	1/30

^†^Allele frequency in this study.

Seven subjects presented new changes in the *CRB1* gene, wherein four novel missense variants were found (p.Leu479Pro, p.Ala921Pro, p.Cys948Arg and p.Asp1031Asn), two frameshift deletions (c.2536_2542del7 and c.3460_3461delTG) and one frameshift indel variant (c.276_294delinsTGAACACTGTAC) (see Table 4). None of them were present in the ClinVar^8^, ESP^9^, ExAC^10^ and 1000 Genomes Project^11^ databases. All new frameshift variants occur in the extracellular domain, leading to premature termination of the protein with the loss of the transmembrane region, contrasting with data found in patient 9 (p.Arg1390*) where protein truncation caused the loss of the PDZ-binding motif but the transmembrane domain was preserved.

**Table 4:** Novel likely-pathogenic variants in *CRB1* gene identified in this study.

Nucleotide Change	Protein Change	Effect	*in silico* Analysis			Pathogenicity
			Poly-Phen2^†^	PROVEAN	SIFT	
c.276_294delinsTGAACACTGTAC	p.Arg92Serfs*54	Frameshift/protein truncation	—	—	—	Likely pathogenic
c.1436 T > C	p.Leu479Pro	Change of highly conserved residue	D	D	T	Likely pathogenic
c.2536_2542del7	p.Gly846Serfs*8	Frameshift/protein truncation	—	—	—	Likely pathogenic
c.2761 G > C	p.Ala921Pro	Change of highly conserved residue	D	D	T	Likely pathogenic
c.2842 T > C	p.Cys948Arg	Change of highly conserved residue	D	D	D	Likely pathogenic
c.3091 G > A	p.Asp1031Asn	Change of highly conserved residue	D	D	D	Likely pathogenic
c.3460_3461delTG	p.Cys1154*	Frameshift/protein truncation	—	—	—	Likely pathogenic

^†^Poly-Phen2 HumVar; D – Probably Damaging; T – Tolerated.

**Figure 3:**
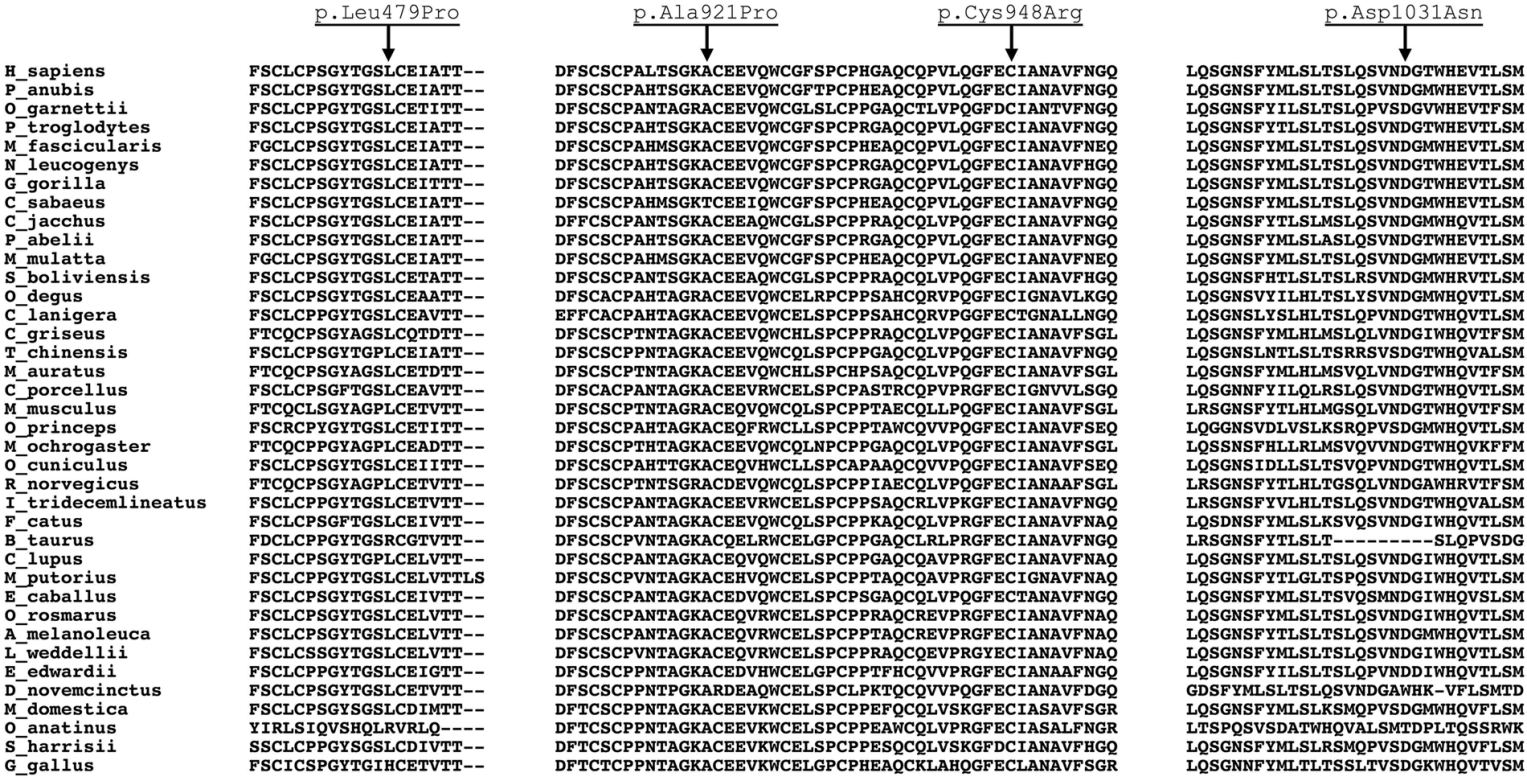
Amino acid conservation analysis of novel missense variants identified in this study.

*In silico* analysis of new missense variants showed that, they were classified as likely pathogenic for at least two predictors (Table 4). In comparison with other species, it is noted that amino acids changed are highly conserved (Fig. 3), mainly among primates (first 12 species of Fig. 3).

All novel variants found in this study can be classified as likely pathogenic according to the criteria of effect in the protein structure, amino acid conservation, frequency in population and classification by pathogenic predictors (Table 4).

## Discussion

Mutations in the *CRB1* gene lead to visual impairment and even complete blindness in individuals with many different clinical IRD phenotypes, including LCA, EORD and RP^6, 12–14^. Despite the large phenotypic heterogeneity among CRB1 patients, some signs, symptoms and ophthalmologic findings can be observed with more frequency^12^. Overall, the subjects in this study presented many of typical characteristics including: nummular pigmentation, macular atrophy, bone spicules, nystagmus and poor central vision in patients with LCA and macular preservation, peripheral bone spicules, pigmentation changes of the RPE, nyctalopia and tunnel vision in typical RP patients.

In two cases (patients 14 and 15), the molecular result was not conclusive because only one pathogenic variant was found. As the inheritance pattern of IRD caused by *CRB1* mutations is autosomal recessive, then the presence of pathogenic variants in both alleles are required for the molecular test to be conclusive. While advances in the molecular diagnosis of IRD are moving fast, the next-generation sequencing still do not solve 35–45% of IRD cases^15–18^.

In the past, negative or inconclusive diagnoses have occurred through the screening of known mutations, such as in patient 15, tested by DNA microarray (APEX). The Sanger sequencing technique subsequently allowed for an improved analysis of specific genes and identification of known and new variants, such was the case of patients 1, 3, 4, 13 and 14.

Sanger sequencing of patient 14 identified, in addition to the pathogenic variant (c.498_506del9 - p.Ile167_Gly169del), a further three heterozygous variants: c.849-35 T > C (rs1337167), c.989-53 G > T (rs2786098) and c.*28 T > C (rs41302107). They are not rare in population databases^10, 11^ and considered likely benign^8, 19^. Conte and coworkers (2015) showed that retinal dystrophy could be caused by mutations in seed regions of miRNA^20^. Therefore, the 3′UTR variant was analyzed in PolymiRTS Database 3.0^21^ and TargetScan v.7.0^22^ to identify whether it could cause changes in miRNAs or in those target regions. The c.*28 T > C variant possibly changed some miRNA binding sites, as shown in Supplementary Figure S4. Changes in miRNA binding sites may affect *CRB1* expression and contribute to the patient’s phenotype. However, the real effect of this change requires further investigation.

Two CRD patients showed likely pathogenic variants in *CRB1* gene. CRD is caused by mutation in many genes such as *ABCA4, ADAM9, C8orf37, CDHR1, CRX, DRAM2, GUCA1A, GUCY2D, PITPNM3, POC1B, PROM1, RAB28, RAX2, RIMS1, RPGRIP1, SEMA4A*, and *TTLL5* ^6^. Genetic reference databases, including OMIM^6^ and RetNet^7^, do not indicate an association between *CRB1* and CRD. Up to now, only three studies have found this causal relationship, with one describing three unrelated subjects^23^, another, a consanguineous nuclear family^24^ and the third, one proband with a novel splice-site mutation^25^. Our data supports the hypothesis that *CRB1* can also cause CRD and thus the *CRB1* gene might be included in target list for CRD genetic testing.

*CRB1* mutations are considered a risk factor in the development of Coats-like vasculopathy^13, 26^, and, because of this, retinal vascular characteristics should be always evaluated in *CRB1* patients. Among 15 patients analyzed in this study, approximately 67% showed vascular abnormalities such as: vascular tortuosity, arteriolar sclerosis, increased vascular permeability and leakage of fluid and blood, which may mean the beginning of a Coats-like disease. An interesting aspect to note is that mutations in *CRB1* can cause osteoclast deposition on top of vessels or in the paravenous region (Fig. 1). It was not possible to associate these vascular phenotypes with a specific retinal dystrophy or specific mutation in these subjects, corroborating data in the literature which states that Coats-like vasculopathy does not develop solely in RP patients^13, 23, 27^.

Bujakowska and coworkers (2012) published an extensive review of *CRB1* cases, showing that exons 7 and 9 have the highest concentration of pathogenic variants, and p.Cys948Tyr is the most frequent of them^13^. Our findings are similar to these, approximately 69% of variants found in this study are located in the exons 2, 7 and 9 and p.Cys948Tyr is also the most frequent in our samples - it was present in 23% of alleles analyzed.

Interestingly, patient 6 has two different missense variants in the same codon (p.Cys948Tyr and p.Cys948Arg). p.Cys948Arg is not described in literature and also it was found in patient 7. Cysteines have an important role in structure and function of proteins, and variations in this residue are highly likely to cause deleterious phenotypes, especially if the mutated cysteine is part of disulfide bonds^28^, as well as in Cys948 in crumbs homolog-1 protein. Mutations at codon 948 affect the correct folding of 14th EGF-like domain^29^. A large number of exonic variants, missense or synonymous, have already been shown to possess disease-causing effects by disrupting the pre-mRNA’s editing process, causing aberrant splicing^30–32^. Both variants were analyzed in Human Splicing Finder^33^, which indicated them as potential alterations of splicing. Perhaps the greatest deleterious effect caused by changes in 948th codon is not due to the exchange of a conserved cysteine but to mRNA processing problems.

Our data shows a striking pattern between mutation type and the patient’s phenotype. Individuals with severe retinal dystrophy, such as LCA, have two variants affecting the protein function or structure most severely (e.g. frameshift changes, premature stop codon formation, aberrant splicing and lack of disulfide bond, due to mutated cysteine). On the other hand, patients with milder IRD have missense variants or in-frame deletions. Patient 9 and 10 with EORD have an intermediate phenotype and genotype, i.e. a missense variant (p.Arg764His and p.Pro836Thr respectively) and a premature truncation (p.Arg1390* and p.Arg92Serfs*54 respectively). Despite the fact that a genotype-phenotype relationship has not been clearly established in previous studies, they also noted that patients with more severe phenotypes, for example macular atrophy, tend to have protein truncation (nonsense or frameshift deletions) and/or p.Cys948Tyr variants^13, 23, 26^.

To establish a genotype-phenotype correlation in *CRB1* patients is not an easy task. There is a substantial phenotypic overlap and variability between *CRB1*-related diseases and a small number of patients with mutations in this gene. Moreover, the phenotypic modulation possibly occurs due to environmental factors and other genetic factors^12, 13, 26, 34, 35^, such as unknown genes, silent variants causing aberrant splicing^36^, deep intronic mutations^36–38^, copy number variations^39^, complex genomic rearrangements^40, 41^, multigenic inheritance patterns, genetic modifiers^42–44^ and regulators of gene expression^20, 45^. In addition, technical limitations, such as uncovered or low-depth regions in NGS analysis, may hinder the correct molecular diagnosis^46^.

Nowadays, molecular diagnoses are strongly orienting clinical practice in cases of IRD, where there is high genotype and phenotype heterogeneity. The more that new mutations are described and new genotypic-phenotypic associations are made, the greater the knowledge regarding these diseases. Our study highlighted a direct relation between phenotype severity and the mutation effect on protein functionality in CRB1 Brazilian patients, contributing to current knowledge about disease-causing variants and supporting the association between the CRB1 gene and cone-rod dystrophy.

## Methods

This retrospective study reviewed 230 medical records of Brazilian patients with IRD assisted at the Universidade Federal de São Paulo and Instituto de Genética Ocular in São Paulo, Brazil between January 2006 and February 2017. The *condicio sine qua non* to include patients was that they must have already performed at least one genetic test for IRD. Table 5 shows the commercial genetic tests performed on each CRB1 patient.

**Table 5:** Type of Genetic Test performed on *CRB1* patients.

Patient	Genetic Test	Number of Genes Analyzed	Test Date
1	Sanger Sequencing Panel	10	2009
2	Next-Generation Sequencing Panel	19	2012
3	Sanger Sequencing Panel	17	2011
4	Sanger Sequencing	1	2011
5	Next-Generation Sequencing Panel	19	2015
6	Whole Exome Sequencing		2015
7	Next-Generation Sequencing Panel	226	2017
8	Next-Generation Sequencing Panel	226	2017
9	Next-Generation Sequencing Panel	19	2013
10	Next-Generation Sequencing Panel	226	2017
11	Next-Generation Sequencing Panel	131	2014
12	Next-Generation Sequencing Panel	131	2014
13	Sanger Sequencing Panel	3	2011
14	Sanger Sequencing	1	2014
15	Arrayed Primer Extension (APEX)	18 (585 mutations/SNPs tested)	2009

In addition to the genetic data, medical history and eye exams were collected. The clinical hypothesis for patient classification of patients was created based on their signs and symptoms, age of onset and fundus features.

The classification of new variants according to pathogenicity was based on the following criteria: variants with the most potential to cause disease are those that result in truncated protein production (premature stop codon and frameshift changes) or missense changes in highly conserved amino acids, as well as a rare frequency variation in genetic population databases and classified as likely damaging by the pathogenicity predictors. The databases consulted were: HGMD^5^, ExAC^10^, 1000 Genomes Project^11^, Exome Sequencing Project (ESP)^9^ and ClinVar^8^. The pathogenicity predictor softwares consulted were: Poly-Phen2^47^, SIFT^48^ and PROVEAN^49^. Combined Annotation Dependent Depletion (CADD) software^19^ was used to evaluate changes in non-coding regions of the *CRB1* gene. The Human Splicing Finder^33^ was used to check possible aberrant splicing. The bioinformatics tools PolymiRTS Database 3.0^21^ and TargetScan v.7.0^22^ were used to evaluate changes in miRNAs or miRNA binding sites.

For amino acid conservation analysis, *CRB1* gene of 38 species was compared. A multiple sequence alignment was built using PRALINE online toolkit^50^, where all previous selected sequences were submitted to multiple alignment using default parameters. The alignment file was open in Clustal X^51^ in order to build alignment figures. The amino acids were classified as: highly conserved (changed in a maximum of three species), moderately conserved (changed in four to six species) and weakly conserved (changed in more than six species).

Nucleotide numbering is based on reference sequence NM_201253, where A of initiation codon (ATG) is the number 1.

The Ethics Committee in Research of Federal University of São Paulo approved this study (CEP: 0415/2016). Written informed consent for the use of personal medical data for scientific purposes and publication was obtained from all patients and/or their legal guardians. In addition, this study was performed in accordance with the ethical standards of the 1964 Declaration of Helsinki and its subsequent amendments.

## Electronic supplementary material

Supplementary Information
