# Supplementary material for: The correlation between CRB1 variants and the clinical severity of Brazilian patients with different inherited retinal dystrophy phenotypes
Source: Sci Rep. 2017 Aug 17;7:8654. doi: 10.1038/s41598-017-09035-1 (PMC5561187; doi:10.1038/s41598-017-09035-1)
Supplement: Supplementary file 1 — Supplementary Information [file 41598_2017_9035_MOESM1_ESM.pdf]

Supplementary Information

Title: The correlation between *CRB1* variants and the clinical severity of Brazilian patients with different inherited retinal dystrophy phenotypes

Fabiana Louise Motta[1], Mariana Vallim Salles[1], Karita Antunes Costa[1], Rafael Filippelli-Silva[2], Renan Paulo Martin[2], Juliana Maria Ferraz Sallum[1]*

[1] Department of Ophthalmology, Federal University of Sao Paulo, Sao Paulo, Brazil

[2] Department of Biophysics, Federal University of Sao Paulo, Sao Paulo, Brazil

*Corresponding Author: Juliana Maria Ferraz Sallum
Rua Botucatu 822; CEP 04023-062 São Paulo, Brazil
Telephone/Fax: +55 11 38422776
Email: juliana@pobox.com

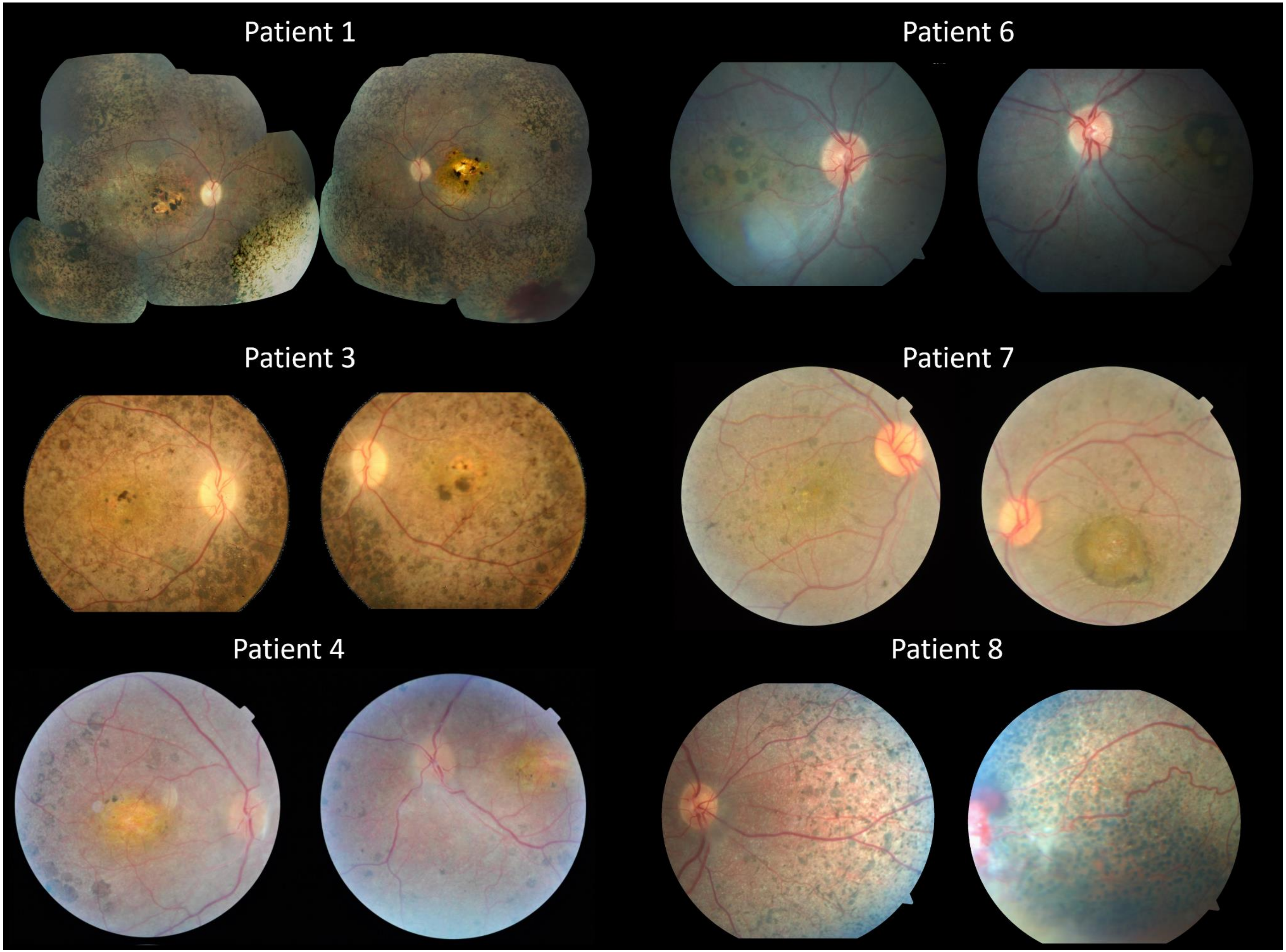

Supplementary Figure S1. Fundus appearance of LCA patients.

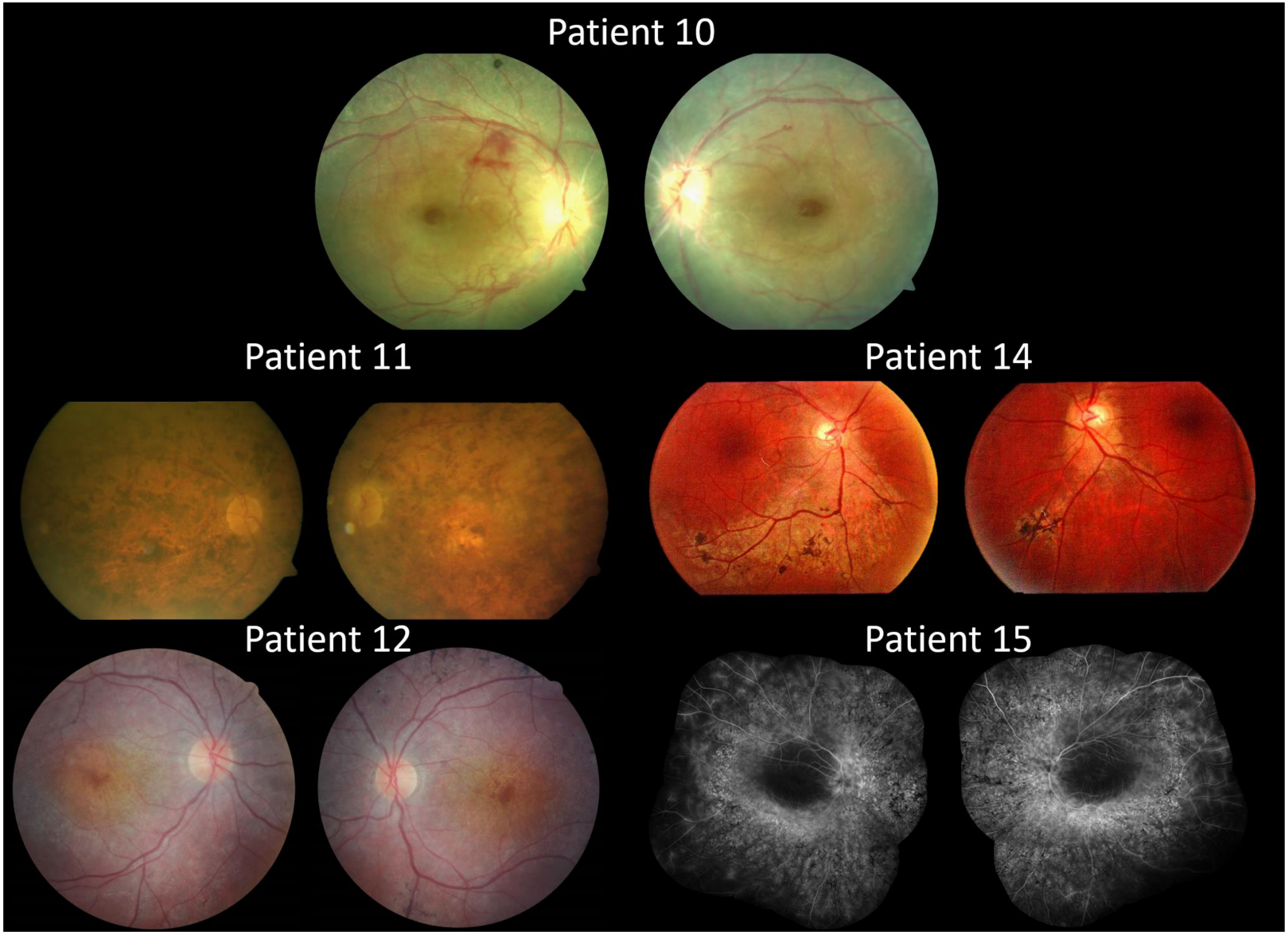

Supplementary Figure S2. Fundus appearance of EORD (10), CRD (11 and12) and RP (14 and 15) patients.

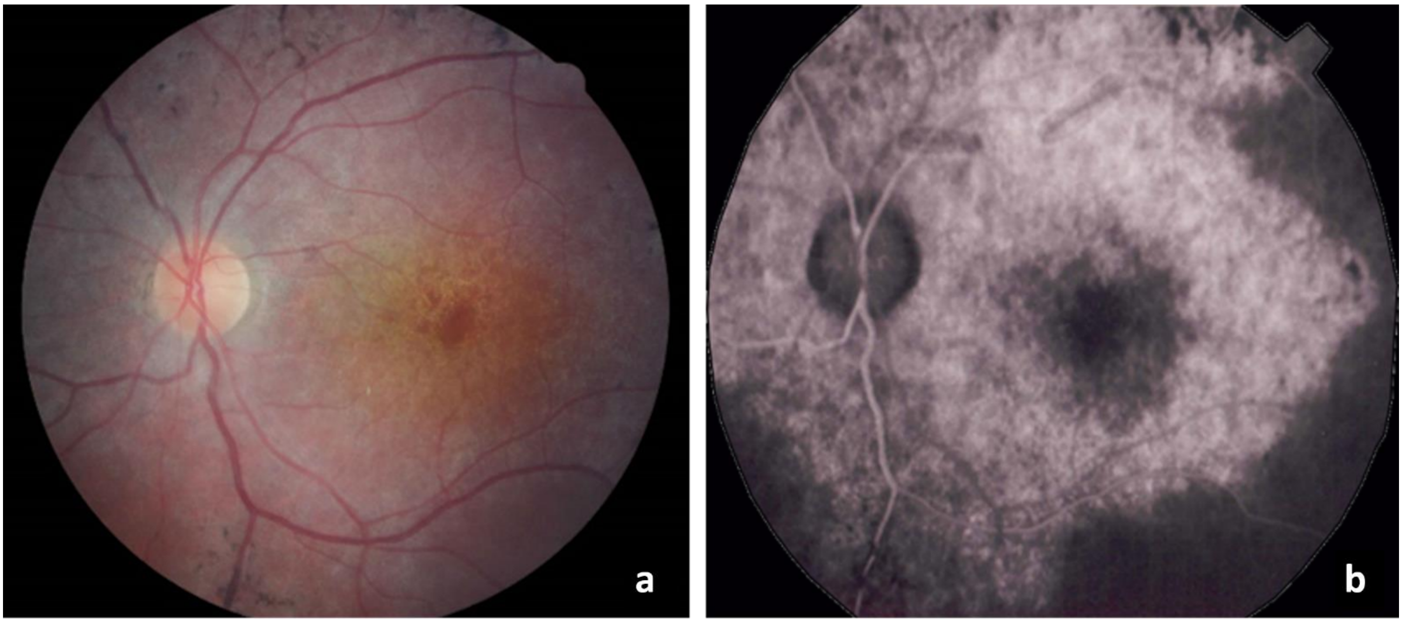

Supplementary Figure S3. Fundus appearance of patient 12. (A) Color fundus photograph showing macular atrophy. (B) Fluorescein angiography showing hyperfluorescence of posterior pole and alteration of RPE pigmentation.

PolymiRTS Database 3.0 - http://compbio.uthsc.edu/miRSNP (Bhattacharya et al. 2014)

RefSeq ID: NM_001193640
Gene Symbol: CRB1
Description: Homo sapiens crumbs homolog 1 (Drosophila) (CRB1), transcript variant 2, mRNA.
Gene Location: chr1(+):197237333-197447585

SNPs and INDELs in miRNA target sites

Location	dbSNP ID	Variant type	Wobble base pair	Ancestral Allele	Allele	miR ID	Conservation	miRSite	Function Class	Exp Support	context+ score change
197447021	rs199582748	SNP	N	C	C	hsa-miR-4475	3	ttgtGTCCCTTcg	D	N	No Change
						hsa-miR-7515	3	ttgtgTCCCTTCg	D	N	No Change
						hsa-miR-7845-5p	4	ttgTGTCCCTtcg	D	N	No Change
					T	hsa-miR-1252-5p	3	ttgtgTTCCTTCg	C	N	No Change
197447037	rs41302107	SNP	N	T	T	hsa-miR-3691-5p	5	tggggATCCACAc	D	N	-0.112
						hsa-miR-376b-5p	5	tggggATCCACAc	D	N	-0.123
						hsa-miR-376c-5p	5	tggggATCCACAc	D	N	-0.123
						hsa-miR-3911	5	tggggaTCCACAC	D	N	-0.234
						hsa-miR-4752	5	tggggATCCACAc	D	N	-0.109
						hsa-miR-8056	5	tggggATCCACAc	D	N	-0.111
					C	hsa-miR-6748-5p	5	tggggACCCACAc	C	N	-0.134
						hsa-miR-6759-5p	5	tggggACCCACAc	C	N	-0.167
						hsa-miR-6793-5p	5	tggggACCCACAc	C	N	-0.144

Function Class: **D**: The derived allele disrupts a conserved miRNA site (ancestral allele with support >= 2).
C: The derived allele creates a new miRNA site.

TargetScan (v7.0) - www.targetscan.org (Agarwal et al. 2015)

Poorly conserved

<table>
<tr><th></th><th>Predicted consequential pairing of target region (top) and miRNA (bottom)</th><th>Site type</th><th>Context++ score</th><th>Context++ score percentile</th><th>Weighted context++ score</th><th>Conserved branch length</th><th>P_{CT}</th></tr>
<tr><td>Position 18-29 of CRB1 3' UTR
hsa-miR-6870-3p</td><td>5' ...UCCCUUCGAGAUGGGGAUAUCCAC...
|||||||||||
3' GACUUUCCUCUACCCCUACUCG</td><td>non-canonical</td><td>N/A</td><td>N/A</td><td>0.00</td><td>0</td><td>N/A</td></tr>
<tr><td>Position 18-29 of CRB1 3' UTR
hsa-miR-6870-3p</td><td>5' ...UCCCUUCGAGAUGGGGAUAUCCAC...
|||||||||||
3' GACUUUCCUCUACCCCUACUCG</td><td>non-canonical</td><td>N/A</td><td>N/A</td><td>0.00</td><td>0</td><td>N/A</td></tr>
<tr><td>Position 27-33 of CRB1 3' UTR
hsa-miR-376b-5p</td><td>5' ...CCCUUCGAGAUGGGG-AUCCACAC...
||| ||||||
3' UUUGUAUCUUCCUUAUAGGUGC</td><td>7mer-A1</td><td>-0.16</td><td>76</td><td>-0.16</td><td>0.031</td><td>N/A</td></tr>
<tr><td>Position 27-33 of CRB1 3' UTR
hsa-miR-376c-5p</td><td>5' ...CCCUUCGAGAUGGGG-AUCCACAC...
||| ||||||
3' UUGUAUCUUCCUUAUAGGUGG</td><td>7mer-A1</td><td>-0.11</td><td>76</td><td>-0.11</td><td>0.031</td><td>N/A</td></tr>
<tr><td>Position 27-33 of CRB1 3' UTR
hsa-miR-3691-5p</td><td>5' ...CCCUUCGAGAUGGGGAUCCACAC...
||||||
3' CAUGGCUCAGAGGUAGUAGGUGA</td><td>7mer-A1</td><td>-0.16</td><td>75</td><td>-0.16</td><td>0</td><td>N/A</td></tr>
<tr><td>Position 27-33 of CRB1 3' UTR
hsa-miR-8056</td><td>5' ...CCCUUCGAGAUGGGGAUCCACAC...
||||||
3' UACGUAGGUCUGUUAGGUGC</td><td>7mer-A1</td><td>-0.07</td><td>59</td><td>-0.07</td><td>0</td><td>N/A</td></tr>
<tr><td>Position 27-33 of CRB1 3' UTR
hsa-miR-4752</td><td>5' ...CCCUUCGAGAUGGGGAUCCACAC...
|||||||
3' UCGUGUAGGAACUCUAGGUGUU</td><td>7mer-m8</td><td>-0.02</td><td>29</td><td>-0.02</td><td>0</td><td>N/A</td></tr>
<tr><td>Position 28-35 of CRB1 3' UTR
hsa-miR-3911</td><td>5' ...CCUUCGAGAUGGGGAUCCACACA...
||||||||||
3' ACGGAGGAGGUCCUAGGUGUGU</td><td>8mer</td><td>-0.20</td><td>88</td><td>-0.20</td><td>0</td><td>N/A</td></tr>
</table>

Supplementary Figure S4. Result of miRNA in silico analysis.
